# Exploration of Brain-Computer Interaction for Supporting Children’s Attention Training: A Multimodal Design Based on Attention Network and Gamification Design

**DOI:** 10.3390/ijerph192215046

**Published:** 2022-11-15

**Authors:** Danni Chang, Yan Xiang, Jing Zhao, Yuning Qian, Fan Li

**Affiliations:** 1 School of Design, Shanghai Jiao Tong University, Shanghai 200240, China; 2 Department of Aeronautical and Aviation Engineering, The Hong Kong Polytechnic University, Hong Kong, China

**Keywords:** brain–computer interface, attention training, attention network, gamification design

## Abstract

Recent developments in brain–computer interface (BCI) technology have shown great potential in terms of estimating users’ mental state and supporting children’s attention training. However, existing training tasks are relatively simple and lack a reliable task-generation process. Moreover, the training experience has not been deeply studied, and the empirical validation of the training effect is still insufficient. This study thusly proposed a BCI training system for children’s attention improvement. In particular, to achieve a systematic training process, the attention network was referred to generate the training games for alerting, orienting and executive attentions, and to improve the training experience and adherence, the gamification design theory was introduced to derive attractive training tasks. A preliminary experiment was conducted to set and modify the training parameters. Subsequently, a series of contrasting user experiments were organized to examine the impact of BCI training. To test the training effect of the proposed system, a hypothesis-testing approach was adopted. The results revealed that the proposed BCI gamification attention training system can significantly improve the participants’ attention behaviors and concentration ability. Moreover, an immersive, inspiring and smooth training process can be created, and a pleasant user experience can be achieved. Generally, this work is promising in terms of providing a valuable reference for related practices, especially for how to generate BCI attention training tasks using attention networks and how to improve training adherence by integrating multimodal gamification elements.

## 1. Introduction

As a rapidly developing research field, the brain–computer interface (BCI) has attracted a lot of attention. It involves multiple disciplines, such as bioscience, neuroscience and information technology, and has become an important research direction. Basically, a BCI allows users to interact with computing systems and realize direct control using brain signals. The neurofeedback capture and analysis can enable the perception and interpretation of brain activity. Widespread applications have been devoted to enhancing cognitive functions for healthy people and the rehabilitation of people with neurodevelopmental conditions [1]. Especially in medical fields, the BCI has become an important tool to assist physically challenged persons with certain mobility and manipulative abilities. Moreover, such capability has shown great potential in facilitating neurofeedback training at home [1].

With the development of consumer-grade devices, the BCI has been increasingly considered in non-medical scenarios, such as supporting non-professional neurofeedback training and entertainment purposes. Recent research also indicates that the BCI has already moved from assistive care to applications in the gaming and entertainment industries, where cheaper and more viable consumer-grade solutions are offered for the general public [2]. According to the different roles of BCIs in these games, they can be classified into games that are only controlled by a BCI and games in which a BCI is used for additional controls [3]. For games controlled only by a BCI, they often rely on neurofeedback to realize game control; for example, the BrainBall game [4] for learning to control relaxation levels represented by occipital alpha intensity, the SSVEP game [5] for improving attention to flickering areas on a computer screen, the BCI-pacMac game [6] for training general control of motor paradigms. Regarding games using BCIs as additional inputs, they mainly rely on a mouse, keyboard or gamepads as input devices and use BCIs to enrich the interaction forms [7]. Most previous studies have mainly focused on technical aspects, such as how to technically develop the brain interfaces and integrate BCIs into games. Recently, researchers have tried to explore more capabilities of BCI applications. For example, data through mental state monitoring by BCIs can help estimate a user’s status, such as their mental workload and focused attention level in real time. Games can use the additional user status information to increase the level of immersion and entertainment [8,9]. These BCI games not only provide a new way of game interaction for normal users, but also enable a new rehabilitation treatment method [10]. Therefore, further theoretical studies to interpret users’ brain activities and evaluate the practical effects caused by BCIs on the game or therapy experience based on more comprehensive and detailed user data are worthwhile [2].

Looking into specific BCI practices, attention training is one important application area. Actually, attention plays an important role in the establishment of children’s learning process [11]. Attention deficiency can result in learning difficulties, poor performance, and even the slow development of intelligence. Hence, many studies have been proposed to train attention via either professional medical interventions [12] or non-medical training [13]. Among them, the BCI is a promising way of training attention. Taking advantage of game-based learning/training to facilitate active learning, participation and interest [9], several BCI games were developed to train the attention of autistic children [14]. In addition, a BCI was used to train attention during stroke rehabilitation [15]. However, “*the major usability and technical challenges existing BCI faced have resulted in the mistrust from healthcare providers, poor treatment adherence and limited adoption”* [1]. Despite the efforts of the BCI suggesting an effective improvement to some attention symptoms, whether consumer-grade BCI headsets and training games can improve attention in the wider population still lacks empirical support.

Through an analysis of existing practices, it can be found that the BCI has been widely considered for supporting children’s attention training and has shown great potential in estimating users’ mental states. However, the existing training tasks are relatively simple and lack reliable task generation and arrangement processes. Moreover, the training experience has not been sufficiently studied, and empirical validation of the training effect is still needed. To tackle these issues, this study intends to further explore BCI solutions for attention training and improvement. Specifically, to overcome the challenges in effectively integrating BCIs into gameplay, the attention network, which regards attention as an independent cognitive network with three subsystems and points out structured references for attention evaluation, is referred to in order to construct the training tasks. To improve the training adherence and experience, the gamification design theory is introduced to guide the gameplay mechanics. To provide solid validation of the empirical effect of the BCI training system, an experimental analysis process is organized to quantitively reveal the influence of BCIs on children’s attention improvement. Therefore, the possible contributions of this work may include:

(i) The development of a new and effective BCI attention training system with reference to attention network theory.
(ii) The integration of gamification design theory for improving the user’s experience and training adherence.
(iii) The demonstration of the efficiency of the BCI training game in improving normal children’s attention ability via a hypothesis-testing experiment.

The remainder of the paper is structured as follows: Section 2 summarizes the state-of-art articles related to the development of the brain–computer interface, the attention network theory for assisting attention training and the gamification design for improving training experience. The specific establishment procedures of the BCI gamification training system are presented in Section 3, consisting of training hardware sets and training games. To validate the training effect of the proposed system, a series of user experiments are organized in Section 4, and the experiment results and primary analysis are presented in Section 5. Further discussion is expanded to reveal the values of this work compared with other attention training works in Section 6. Section 7 concludes the study and points out the future research directions.

## 2. Related Work

In this section, the development of brain–computer interaction is summarized, and the potential of BCIs for assisting non-medical training is revealed. Subsequently, the attention network theory, which presents important reference meaning for constructing effective attention training and evaluation, is introduced. To further consider training adherence, gamification design, which is helpful in improving the training’s attractiveness and the user’s experience, is presented. Based on the analysis of existing practices, it was identified that BCI games have a huge potential to facilitate the non-medical attention training process.

### 2.1. The Development of Brain–Computer Interaction

With the ability to understand brain states to communicate with machines, the brain–computer interface (BCI) has received considerable attention in the last few years [16]. Research on BCIs began in the 1970s. Most studies have concentrated on using BCIs for controlling prosthetics, rehabilitation and interfaces for users with motor disabilities. Neuroprosthetic devices implanted in animals and humans have demonstrated remarkable research success in “reading” the brain to detect expected movements and using extrapolated signals to move robots or prosthetic devices [17]. Therefore, conventional BCIs have usually been aimed at assisting, augmenting or repairing human cognitive or sensory–motor functions [16].

Generally, there are two types of methods to extract signals from the brain, i.e., invasive BCIs, and non-invasive BCIs. For invasive BCIs, they are implanted directly into the brain. Using chips implanted within the brain, scientists are able to read the firing of neurons in the brain. The invasive devices can deliver BCI signals of high quality [18]. However, over time, scar tissue tends to form, which leads to signal loss [19]. Additionally, the implanted sensors cannot be moved to measure the rest of the brain [20]. Non-invasive BCIs are means of reading brain activity from the surface of the skull, rather than making direct neural contact via pins [18]. The signal acquired by non-invasive techniques has a low signal-to-noise ratio. Electroencephalography (EEG), magnetoencephalography (MEG), functional magnetic resonance imaging (fMRI) and functional near-infrared spectroscopy (fNIRS) all provide non-invasive neuroimaging techniques [21]. These methods work differently, providing different levels of portability, and spatial and temporal resolution [22].

Although initially BCI research was focused on applications for paralyzed patients, an increasing number of alternative applications have been proposed and studied in the health care area [23]. Brain activity can be interpreted by both invasive and non-invasive monitoring devices, thus providing novel therapeutic solutions for non-medical applications [24]. In this regard, researchers made some explorations including the monitoring of mental states such as performance capability or task engagement in industrial applications [25], the assessment of music influence on users’ cognitive abilities in various contexts [26], the realization of more seamless interactions between humans and artificial cognitive agents [27] and the real-time classification of brain states based on users’ cognitive workload, multitasking, preference detection and so on [28]. These applications demonstrate the great potential of BCIs for non-medical use. Especially with the development of consumer-grade brain–computer devices, more possibilities are being presented for applying BCIs in casual scenarios [3]. Such devices cost less and provide open-source interfaces, and the wireless connection makes users feel more comfortable in wearing them and make them more convenient to use [29]. Therefore, utilizing BCIs to generate effective solutions for non-medical purposes is a promising direction.

### 2.2. Attention Network Theory for Structured Attention Analysis and Evaluation

During the practical use of consumer-grade BCIs in attention training, the lack of the effective organization of the training process can lead to the mistrust issue. For this problem, cognitive theories and methods have been widely studied to provide solid evidence for the training process. Attention network theory was proposed by Posner based on many experimental studies in cognitive psychology and biological science in 1990 [30]. It regards attention as an independent cognitive network system. In this way, attention can be directed independently of and concurrently with other operations. Considering the multifaceted nature of attention, three subnetwork systems with different functions were proposed: the alerting network, the orienting network and the executive network [31].

Specifically, the alerting network has the property of keeping individuals in an optimal state of alert in order to respond quickly and accurately to incoming stimuli [32,33]. The orienting network is responsible for selecting specific information from outside in a sensory pathway [31]. The executive network encompasses many functions, and is mainly responsible for resolving reaction conflicts, decision making, self-regulation and flexibly adjusting behaviors according to task requirements, and also involves resolving conflicts in different tasks.

Based on the attention network theory, Fan et al. [34] developed an Attention Network Test (ANT) to evaluate the performance of three attention networks within a single 30-min testing session. It is easily performed by children, patients and monkeys, and has been useful in assessing attention abnormalities associated with brain injury, stroke, schizophrenia and attention deficit disorder. The test requires participants to concentrate, react quickly after cues and press the correct arrow keys according to the direction of the target arrow [33]. In the original ANT, the effect of the three networks is evaluated by the average reaction time (RT) of the subjects under different cues:

The effect parameter of the alerting network = RT (no cue) − RT (double cue), and the difference between the presence and absence of cues reflects the efficiency of the alerting network.

The effect parameter of the orienting network = RT (center cue) − RT (spatial cue), and the difference between the presence or absence of spatial cues reflects the efficiency of the orienting network.

The effect parameter of the executive network = RT (incongruent) − RT (congruent), and the difference in the interfering stimuli accompanying the appearance of the target reflects the efficiency of the executive network. 

In subsequent years, a multitude of other variations to the ANT have been developed. For example, Rueda et al. developed ANT-C (Child ANT) to assess the attention network of children [35]. This ANT provides more appealing and visually stimulating variants that will be more fun for children. Later, Lateralized ANT (L-ANT) was developed by Greene et al. [36], which presents the target in a horizontal plane rather than a vertical plane and determines that each hemisphere in the brain can support the attention network. In addition, other variants of the ANT, such as the revised ANT (ANT-R) [34] and the vigilance ANT-I (ANTI-V) [37], have been developed.

Attention network has been widely studied and developed in the past years, and most of the research objects have been patients with depression and insomnia. Compared with other cognitive theories and methods, ANT is more practical and instructive for attention training and evaluation. However, the current ANT practices are mainly based on repeated tasks, which are relatively boring. Novel and interesting forms are expected to continuously improve users’ sense of immersion and participation [38]. Especially for the attention training for children, attractive training tasks are particularly important. Therefore, the combination of the ANT system and BCIs is promising to provide attractive tasks based on children’s characteristics in cognition, behaviors and expectation.

### 2.3. Gamification Design for User Experience Enhancement

Regarding existing BCI practices, tasks are often repeated and lack enough attractiveness to ensure proper treatment adherence. To tackle this problem, related efforts have been devoted to facilitating gamification to improve the treatment experience. Gamification is defined as “the use of game design techniques in non-game contexts” to improve user experience and user engagement [39], and has been widely researched for several years. Especially in an education context, it has been demonstrated that gamification can greatly increase students’ learning motivation and facilitate the learning effect [9]. Similarly, practices in other areas show that gamification can effectively improve user experience, and increase user acceptance, motivation and participation [40,41]. Compared with the traditional game mode, multimodal gamification can further enhance users’ pleasant experience and provide effective support for user interaction [42]. Basically, there are several classical gamification frameworks, including Six Steps to Gamification presented by Werbach and Hunter [43], GAME proposed by Marczewski [44], a Back to Contents Gamification design process based on human–computer interaction (HCI) principles defined by Marache-Francisco and Brangier [45], a complete gamification framework called Octalysis proposed by Yu-kai Chou [46], and “A Framework for Sustainable Gamification Impact” presented by AlMarshedi et al. [47], etc. Among them, the Octalysis framework has been commonly adopted, and has proved successful in bridging game elements and play behaviors.

The Octalysis framework was first developed by Yu-kai Chou with the idea that individual actions are motivated by eight core drives [48]. Specifically, the eight core drives are proposed as follows [42,49]:

(1) Epic Meaning and Calling: the core drive about selfless acts, where a person believes that they are doing something greater than oneself or they were “chosen” to take action.
(2) Development and Accomplishment: the internal drive of people to always make progress, develop skills and eventually accomplish a goal.
(3) Empowerment of Creativity and Feedback: the core drive focusing on people’s creative activity where they repeatedly figure things out and try to find out different combinations.
(4) Ownership and Possession: the drive that comes up when users are motivated because they feel like they own something to control as well as trigger the eagerness to improve it.
(5) Social Influence and Relatedness: the drive that covers all the social elements that motivate people, including mentorship, acceptance, companionship, competition and envy.
(6) Scarcity and Impatience: the drive of wanting something that is rare, exclusive or not immediately attainable.
(7) Unpredictability and Curiosity: a drive of constantly being engaged because people do not know what will happen next.
(8) Loss and Avoidance: the core drive that is based on the motivation to avoid something negative.

The main benefit of this framework is the direct connection between the core drives and specific game mechanics’ construction [50]. In recent years, the Octalysis framework has been widely referred to in game development [42]. To improve the users’ experience and users’ acceptance of the attention process, the gamification approach might be a viable solution to embed game elements in training tasks. Therefore, the Octalysis framework has an important reference meaning for assisting the gamification of the attention training process.

Based on the analysis of related practices, it can be found that there is a clear trend when exploring BCI games in different application areas. The Octalysis framework combines the advantages of BCIs of enriching the interaction forms and realizing game control directly by brain signals [51] with the advantage of gamification that provides attractiveness, which can greatly improve the task accomplishment channels and training adherence. Moreover, the multimodal interaction brought by a BCI game can greatly enhance a user’s enthusiasm, improve a user’s acceptance, assist in the effective execution of related tasks and lead to the precise detection and estimation of the user’s state [52]. It has been claimed that the combination of HCI and multimodal gamification can improve the utilization, development and support of human cognitive abilities, and result in more creative and innovative ways of thinking and knowledge acquisition [53]. Owing to these benefits, it can be anticipated that the application of BCI in attention training is promising. However, the existing training tasks are still designed simply, and further improvement in training mechanics with better user experience is still needed. Moreover, current studies are mostly application-driven, and solid empirical validation on the practical effects of BCI games on attention training is still needed. Specifically, a series of research hypotheses were established to help with the systematic examination of the BCI attention training effect. To avoid the bias caused by the subjects, it is examined whether the subjects involved have a generally similar attention performance before the experiments. Therefore, the first hypothesis is H1: There is no significant difference between subjects in attention behaviors before BCI training. According to the literature review, the BCI has shown great potential in attention training. Hence, H2 can be generated: The attention ability of subjects taking BCI training will improve significantly. Without suitable interventions, children’s attention ability should be stable. Hence, H3 can be generated: The attention ability of subjects taking no BCI training will not significantly change.

In the following sections, a BCI attention training system is developed referring to the attention network and integrating gamification design elements, and user experiments are organized to examine the practical training effect of the proposed system through a hypothesis-testing approach.

## 3. Establishment of BCI Gamification Training System

**Figure 1:**
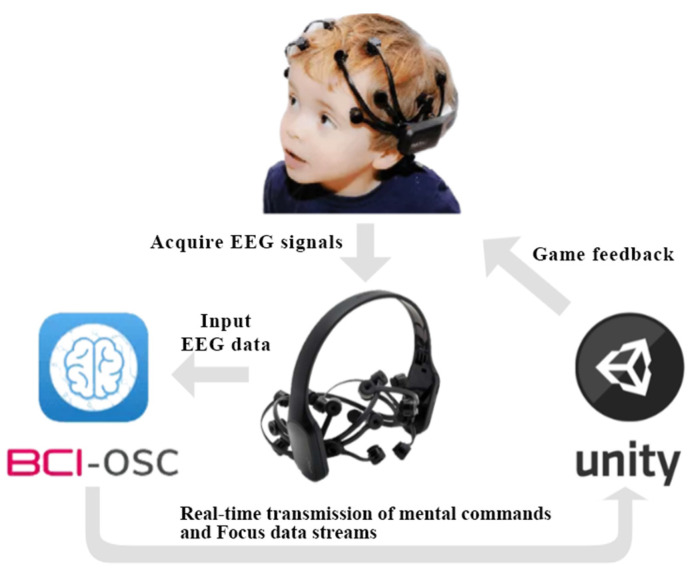
The overall framework of the training system (authors’ proposal).

In this section, the BCI attention training system based on ANT and gamification is developed. In particular, two subsystems are constructed, including the hardware system, which detects the brain signals, and the software system, which mainly includes a training game referring to ANT and the Octalysis framework. in particular, consumer-grade brain–computer devices are employed to detect the EEG signals from users. The data are then transmitted to data analysis software, which allows the retrieval of brain wave data by third-party applications or devices and conducts the data processing, data filtering and basic calculations. With the brain signals inputs, the game development tool of Unity is used to realize the training game. The connections between the hardware and software are presented in Figure 1.

**Figure 2:**
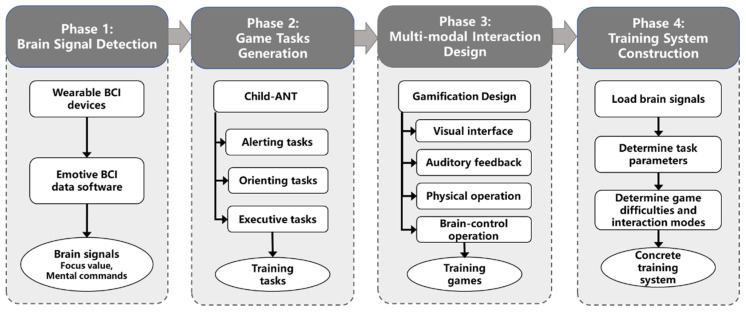
The detailed development procedures of the training system (authors’ proposal).

The detailed development procedures of the proposed training system are summarized in Figure 2. Phase 1 is to configure the hardware settings to detect and collect the brain signals. Phases 2 and 3 are to construct the training games. With the data captured by BCI devices and the training games, the whole training system can be built.

### 3.1. Training Hardware: BCI Devices

**Figure 3:**
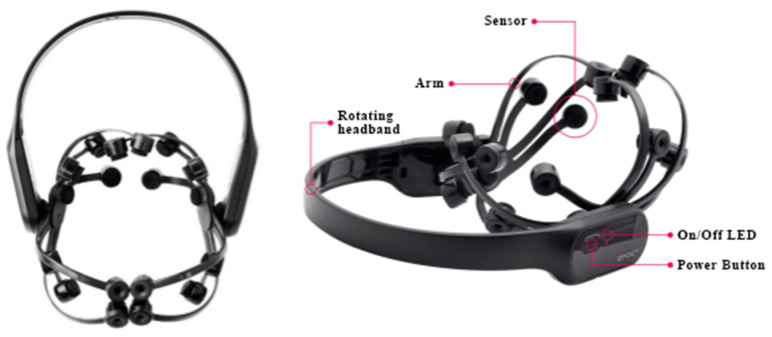
Consumer-grade brain–computer device Emotiv Epoc X.

In this work, the brain–computer device Emotiv Epoc X (as shown in Figure 3) was selected by jointly considering the detection precision, and the data privacy and security, which need to meet the relevant requirements of neuroethics, and the wearing convenience. Epoc X has a total of 14 EEG data acquisition sensors, which can ensure the collection of necessary EEG signals. Considering that EEG signals are in many types and the signal filtering and processing is very complex, it also provides a variety of built-in data measurement toolkits, which can estimate the user performance based on basic EEG data. Therefore, the performance metrics, such as attention (expressed as Focus), facial expressions and mental commands (towards left or right, etc.), can be directly obtained and used by third-party applications. To guarantee the estimation accuracy, preliminary training is often needed to build user training data and improve the estimation precision.

**Figure 4:**
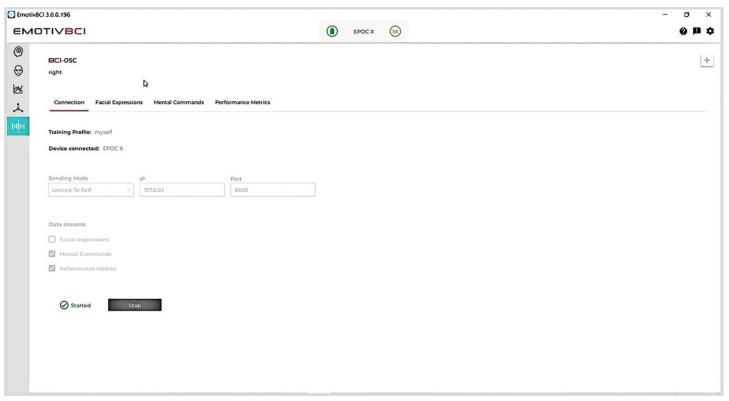
The data analysis software of EmotivBCI.

To support better understanding, the data analysis software EmotiveBCI was also introduced. The main interface is shown in Figure 4. The EEG data collected by Epoc X can be transmitted to the software. In the Open Sound Control (OSC) section, the link between the device and the software can be configured under Connection. In addition, Facial Expressions, Mental Commands and Performance Metrics are provided. Once selected, the related data are retrieved and the real-time data can be transmitted to connected applications. For this training system, the values of Performance Metrics (including the Focus value, which can embody the stable concentration status) and the Mental Commands (toward left or right, which can be easily embedded in game commands) are mainly considered as control inputs.

### 3.2. Training Software

In this section, the gaming mechanics are designed and developed referring to Child ANT and the gamification framework. In particular, the systematic training structures of ANT in alerting, orienting and executive aspects are referred to so that the attention can be trained more scientifically. The gamification framework provides the basis for the design of multimodal game elements that can ensure good quality in terms of the user’s interaction and experience. The details are presented below.

#### 3.2.1. Game Development Platform

The training game was developed via the Unity (commonly known as Unity3D) platform, which is a game engine and integrated development environment (IDE) for creating interactive media [54]. This platform provides a complete set of software solutions, which can be used to create, operate and realize any real-time interactive 2D and 3D content [55,56], so it was chosen as the game development platform for this training software. Generally, the training game prototype was assembled using Unity’s UI components, and the Animator component helped to realize the control of game animation. With the basic game materials set up, the main functions of the game were developed.

The connection with the Emotiv device can also be realized in Unity based on the Emotiv OSC data package. The data package includes EEG commands, performance indicators and facial expressions. In this work, only the EEG commands related to game functions (e.g., mental commands for Neutral, Left, Right, Focus value) were used. Taking the data transmission of the EEG command Left as an example, the Boolean function is used. If the tracked EEG data (retrieved from the EmotiveBCI) is greater than the defined threshold, the mental command value sets as “true”, which triggers the corresponding interactive operation, for example, lifting a magic stick to the Left direction. Otherwise, the mental command value sets as “false”. In this work, the threshold value of mental command was set to be 0.3f as the debugging results show that the threshold 0.3f can effectively distinguish the two EEG command states in the game. In this way, the EEG data from EmotiveBCI were loaded as inputs to determine if related commands were triggered or not, and to directly control the game process. Meanwhile, the real-time BCI data were recorded for further analysis and evaluation.

#### 3.2.2. Game Mechanics Based on ANT

**Figure 5:**
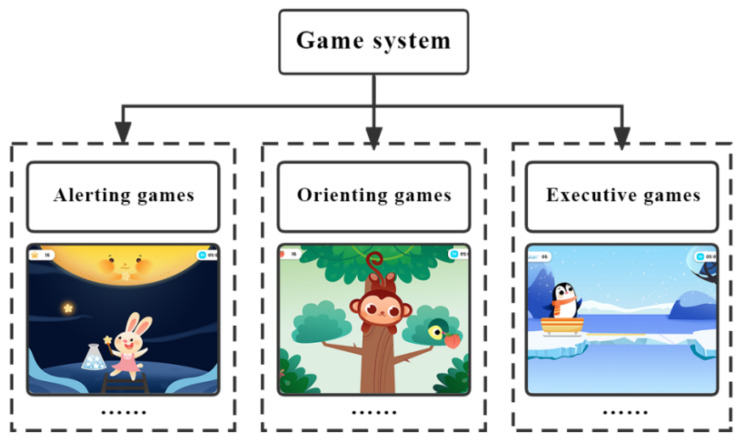
The structure of the game system (authors’ proposal).

The game was constructed based on attention network theory, including three types of games mapping to the alerting, orienting and executive training networks (as shown in Figure 5). Users can choose the proper game type and difficulty level for targeted training according to their own situation. The goal of attention improvement is achieved by repeatedly completing the game training tasks associated with the attention network.

To be interesting to children, the overall game background is set in a unified game worldview, where players explore the animal planet, and each small animal has its own dreams and tasks to be achieved. Helping the animals successfully finish the mission can obtain their favorability. After repeated training, the favorability can be continuously improved to unlock the reward cards and enrich the player’s game book. The unified story background setting is conducive to enhancing children’s sense of participation and acquisition, reducing the monotony of repetitive tasks and improving children’s training adherence.

As mentioned in Section 2, different versions of ANT have evolved. They differ in the event sequence and time interval settings. Considering that this design is for children aged 4–6 years old, the main reference is Child ANT [57], which provides clear settings of the event sequence of the fixation, cue, rest, target and feedback stages. 

**Figure 6:**
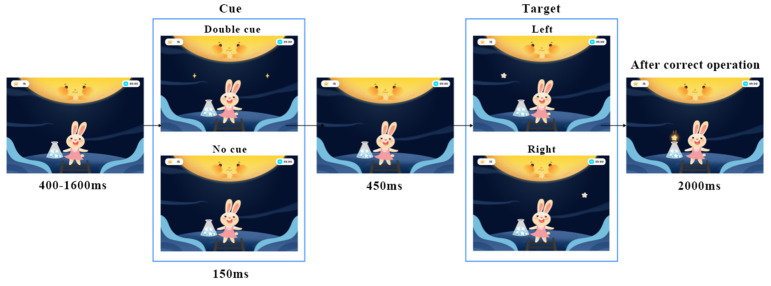
The sequence of events of the alerting game (authors’ proposal).

Taking the alerting game as an example, the story scenario is that the rabbit is on a trip to the moon, and it needs to collect enough stars for landing on the moon. Only when enough stars are obtained can the galaxy bridge be successfully built and help the rabbit land on the moon. Referring to ANT-C, the game mainly includes command judgment and maintaining attention. In the command judgment, the user needs to use mental commands or traditional touch screen interaction to complete the left or right judgment according to the position of the stars, triggering the rabbit to raise the magic stick. Before the stars appear, two cue modes may appear randomly: doubles cues (diamond patterns appear on the left and right of the screen at the same time), and no cues. The ratio of the two kinds of cues to appear is 1:1. In the maintaining attention stage, the user needs to concentrate to make the Focus value reach the corresponding threshold to gradually lighten the stars. After maintaining attention for 2000 ms, the lightening is reached, and the collection of stars is completed. Stepwise game design makes it easier for users to understand and immerse themselves in the game. The duration of each game task is limited to five minutes to protect children’s eyesight and ensure effective training. The game event sequence and interaction control steps are shown in Figure 6.

**Figure 7:**
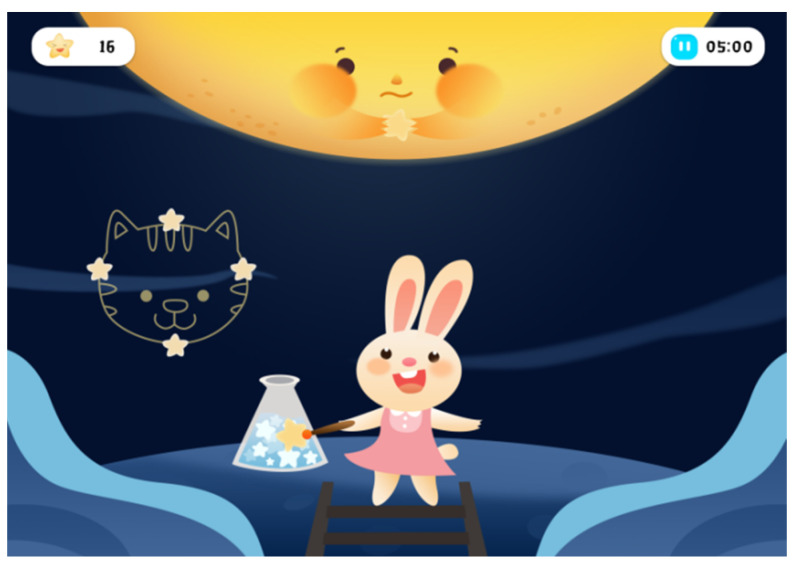
Higher difficulty of the alerting game (authors’ proposal).

The training difficulty increases with the training progress. The user needs to maintain their attention for a longer time and light up the figurative patterns composed of different numbers of stars to complete the task (as shown in Figure 7).

**Figure 8:**
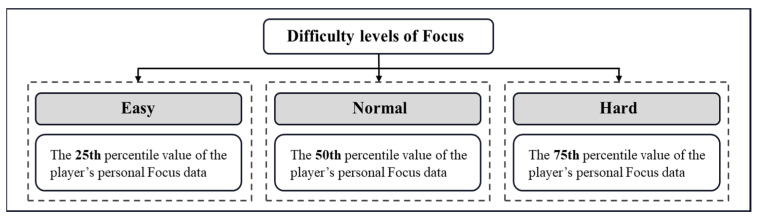
Attention value thresholds for different difficulty levels.

Regarding the Focus thresholds for different difficulties, three difficulty levels are specified. Since the Focus value varies with each person’s attention ability, it is difficult to assign a constant value to them. In this work, pretraining was arranged to collect the attention range of a group of users, and the Focus values of the 25th percentile, median and 75th percentile were set as easy, normal and hard levels, respectively (as shown in Figure 8). Only when the corresponding attention level is reached, can the attention tasks be completed.

**Figure 9:**
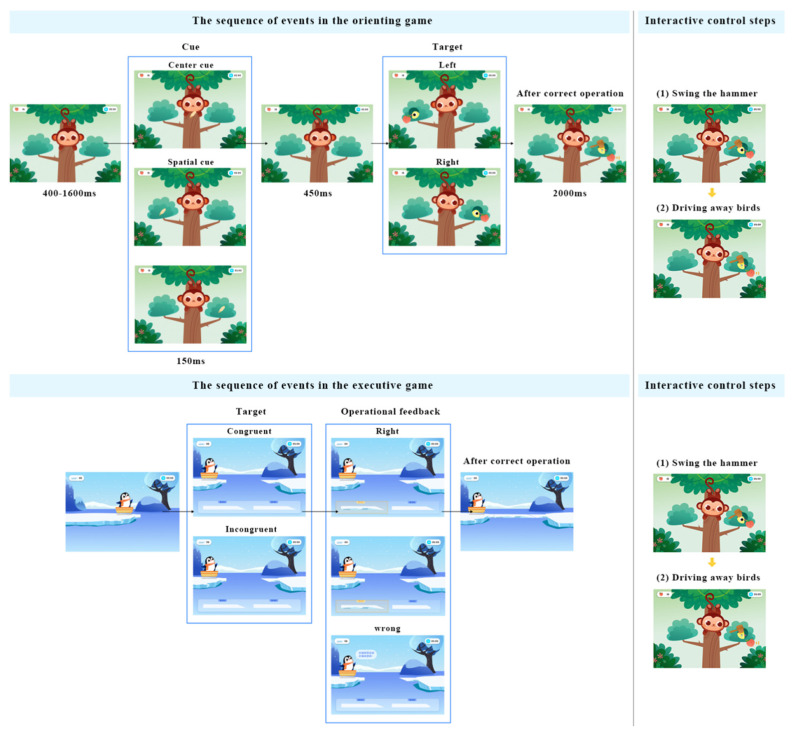
Basic ANT–based game mechanics of the executive and orienting games (authors’ proposal).

Compared with the alerting game, the settings of the orienting game and the executive game are different, but the game mechanics include similar command judgments and maintaining of attention. The event sequence and related interaction prototype diagram are shown in Figure 9.

#### 3.2.3. Multimodal Interaction System Based on Gamification Design

In this section, the core drives of the Octalysis framework are incorporated to direct the design of multimodal interaction elements.

##### Interaction Modes for Game Operations

Development and Accomplishment is the intrinsic drive for people to progress, learn skills, master proficiency and overcome challenges [48]. In this respect, the design of the “challenges” is very important. Considering the psychological and physiological development situation of children, brain–computer interaction may need a certain learning cost for them to realize effective control. If they are frustrated at the initial stage, it will be very hard to motivate them again. Therefore, two interaction types are prepared for children: BCI interaction and traditional interaction (e.g., click the mouse, touch screen).

The results of research on interactive products for preschool children also point out that children’s interaction should be based on familiar and conventional actions, such as pointing, dragging and sliding [58]. It is necessary to design easy-to-operate and relatively simple interaction methods to reduce complex gesture operations that do not conform to their developmental characteristics. Therefore, it is important to develop an interactive way with proper operation difficulty to help children become quickly familiar with the game system, and also have the opportunity to further challenge themselves. For this purpose, the combination of BCI and traditional interaction is adopted.

**Figure 10:**
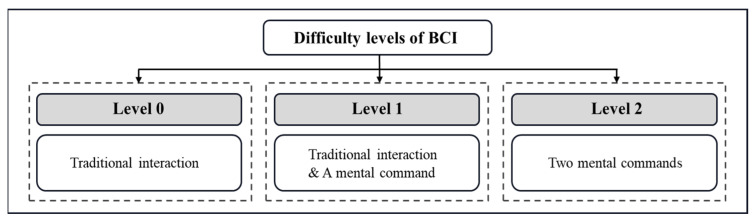
Difficulty levels of the operation interaction modes.

According to the different combinations of interaction modes, the interaction difficulties are divided into three levels (as shown in Figure 10). Level 0 means that traditional interaction is selected to operate the game (i.e., mouse click to trigger the task), and player’s Focus values are just used to finish related tasks (i.e., maintain their attention for reaching the Focus threshold to finish the task). Level 1 means that traditional interactions and one-direction BCI mental commands are used (i.e., once receiving the cues that appear in one certain direction, a mouse click/screen touch is needed to trigger corresponding tasks, and once the cues appear in another direction, a BCI mental command is needed to trigger related tasks). Level 2 supports two-direction mental commands (i.e., both directions, left and right, need proper BCI mental command values to trigger) and totally uses BCI signals to control related game functions. Children can select their comfortable interaction difficulty and increase the interaction difficulty after a period of training.

##### Visual Interface

According to the core drive of Ownership and Possession proposed in the Octalysis framework [48], a visual interface that fits children’s tastes and matches their physiological development stage can make children feel more likely to own the character and have a more immersive experience. If the user has a sense of ownership, it is natural to be motivated and immersed. However, excessive use of visual elements may lead to a negative impact on children’s eyesight, so it should be well designed in terms of visual interaction.

**Figure 11:**
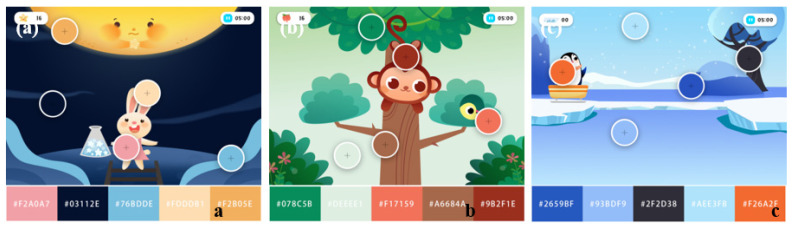
Visual interfaces for different game scenarios. (**a**) Visual interface of the alerting game. (**b**) Visual interface of the orientation game. (**c**) Visual interface of the executive control game. (Authors’ proposal).

Color is an important visual element that captures our visual attention and affects children’s perception of the outside world. In this regard, corresponding strategies are proposed for rich color matching, the reasonable use of bright colors, establishment of a clear color hierarchy and distinguishing game elements that require visual attention from the game background and secondary elements in color use. Moreover, to protect children’s eyesight, too bright colors should be avoided. Specifically, single cool colors are preferred for the background in order to establish a clear visual hierarchy and effectively attract children’s visual attention. The main characters and important elements of the game are designed with warm colors to be clearly distinguished from the background. In addition, the color scheme varies according to the game scenario, as detailed in Figure 11.

##### Auditory Interaction

Referring to the core drives of Empowerment of Creativity and Feedback and Development and Accomplishment in the Octalysis framework [50], the proper game incentive system and feedback loop are expected to motivate the participants to continuously upgrade, enable user-generated content for better game creation and progression, and induce their curiosity and enthusiasm. For this purpose, multichannel interaction is preferred. With the rich sensory stimuli, the training cues can be better communicated, and it is possible to create more interesting training environments and further encourage users’ participation in high quality training. As well as visual interfaces, audio feedback is one important sense that can provide direct instruction and training responses for users. It can stimulate children’s auditory nerves, enrich the sensory experience and train auditory discrimination ability and reaction ability.

**Figure 12:**
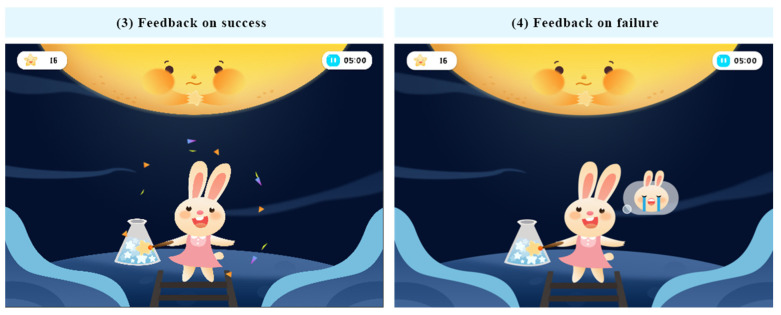
Examples of the training incentives and feedback (authors’ proposal).

**Figure 13:**
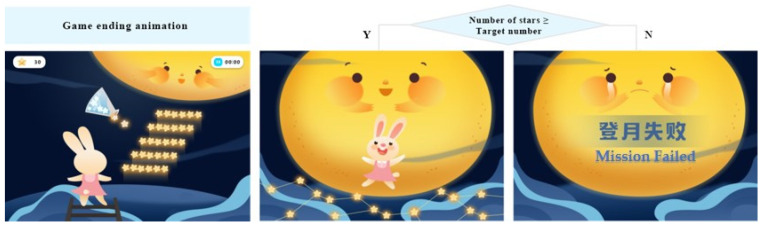
Examples of the feedback animation after one round of training (authors’ proposal).

In this training system, the unified operation sound is provided. The sound effects are assigned for clicking, task completion, reminding of inattention situation, etc. In particular, pure music is preferred for the sound effects since pure music does not increase other information interference and is conducive to children’s immersion in the game. To illustrate specifically in the training system, when the user successfully finishes the training task, the animation of the color bar fragments appears on the game screen, and the audio “Great” is played as positive feedback. When the user fails the task, the rabbit’s crying animation appears until the operation is completed correctly (as shown in Figure 12) and the audio of encouragement is given. After one round of training, the ending animation is played according to whether the training goal has been reached (as shown in Figure 13). With the help of visual and audio interactions, the training incentive and feedback can be better conveyed to participants.

With the training mechanics referring to the attention network and the multimodal interaction design following the gamification principles, the training system can be established. It is expected to provide systematic training tasks and deliver a better training experience. To examine the empirical training effect, evaluation experiments were organized.

## 4. Evaluation Experiments of the BCI Attention Training Game

In this section, the training effect of the developed system is evaluated. In particular, a set of preliminary experiments were organized to build EEG training data sets. The training data were referenced to define the thresholds of Focus values, which were accordingly used as the trigger of the training tasks. Based on the calibration by the preliminary training, formal experiments were performed. The subjective training experience and objective attention ability were assessed through Game Experience Questionnaire, Corner scale and Schulte Grid Test. The experiment was approved by institutional review board (IRB) of Shanghai Jiao Tong University. The details are presented below.

### 4.1. Preliminary Experiments

Since the game involved the use of brain–computer interaction and the capture of EEG data, it was necessary to provide basic training to help users become familiar with the game operations, collect their attention data and determine the attention thresholds for each difficulty level. Therefore, a group of 5 children, aged 4–6 years old and with tablet game experience but no BCI experience before, were recruited to participate in the preliminary experiment, complete the training of mental commands and perform three games with different difficulty levels.

The preliminary experiment lasted for two days. On the first day, a brief introduction of the experiments was given. Having the informed consent from the participants and their parents, basic training was arranged for collecting their EEG data. Taking the alerting game as an example, the participants were trained with the mental command first, and then played the games with different interaction modes (i.e., traditional interaction, one-way BCI and traditional interaction, two-way BCI). All their game performance was observed and recorded. On the second day, the specific parameters of different difficulty levels were adjusted according to the game performance data of the first day. The children were required to play the three games again in a random sequence. After completing one game, they were given a 3 min rest. Similarly, all participants’ attention values in the experiment were recorded. The data during the two-day training were analyzed to determine the proper task difficulties for the formal experiments. Moreover, the children and their parents were interviewed after the experiment to better understand their experience with the training system. These comments were considered to adjust and improve the game settings.

The specific processes of training data establishment and Focus thresholds determination are explained below.

#### 4.1.1. Establishment of EEG Training Set

Effective training before the formal game can facilitate precise interaction operations. Especially considering that many variances exist in the EEG signals among users, a special training set is needed to provide general references for determining the attention level for task control.

**Figure 14:**
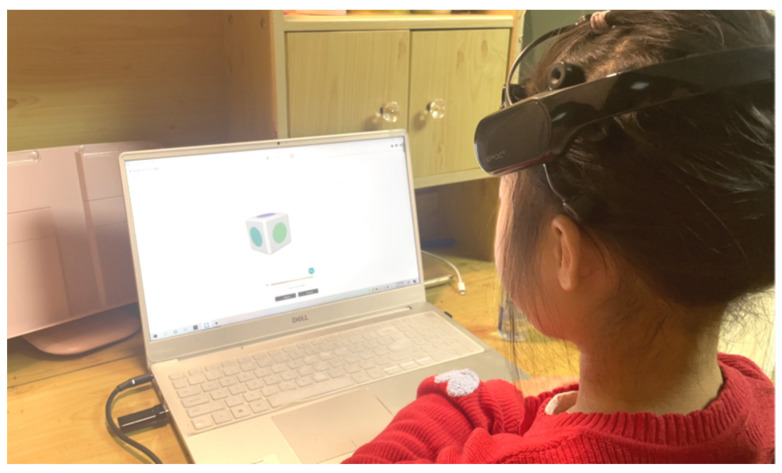
EEG command training.

Specifically, the children were instructed to complete two training states: a neutral state and an attention state (as shown in Figure 14). In the neutral state, the player just remained relaxed. In the attention state, two attention commands were trained, including “Left” and “Right”. Players had to imagine different patterns of brain activity in response to the two commands. They could choose to use motor imagination (imagine objects moving towards the left and right) or image thinking (generate fixed graphics in the mind to build the associations between thinking and attention instructions). Only if the training quality reached the “Great” level or above was the related EEG value effectively detected, the training regarded as effective and the performance recorded as training data.

#### 4.1.2. Determination of Focus Value Thresholds

Focus value is the data that EmotivBCI measures and outputs in real time. Based on the original EEG data detected, internal algorithms are applied to estimate the general focus performance. Since individual differences often exist, it is difficult to assign a constant value as Boolean judgement (true or false) to determine the attention state. Therefore, the preliminary experiments record participants’ attention range (all the subjects’ highest and lowest attention levels), and varying attention levels can be specified accordingly [59]. For example, a low attention level can be set as 25th percentile of the attention range, a medium attention level is the median, and a high attention level reaches the 75th percentile of the attention range. These attention levels are also used as task trigger thresholds for different task difficulties.

**Figure 15:**
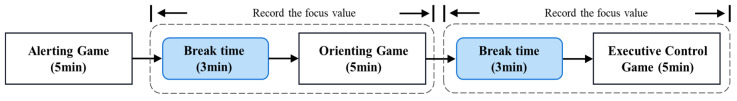
Recording time of Focus value measurement.

The experiment process is presented in the figure below. Participants were required to try the three games. To avoid heavy user burden, every game session was within 5 min. To reduce the influence by the previous game on users’ attention state, a 3 min rest was assigned between different games, and a black screen (with no information or pictures) was presented. In data recording, a 3-min rest and a 5-min game were treated as a complete session (see Figure 15), and participants’ Focus values during each session were captured fully. Considering the system bias, every participant tried the games twice, and it was found that there was no significant difference between the two rounds of experiments. Therefore, the average of the two trials was taken as the final results. Based on the results of all participants, their average attention values were obtained, and a general attention range was determined.

Through the preliminary experiments, it was found that the five children were able to correctly use the brain–computer interaction. They showed high enthusiasm for these novel interaction modes and were willing to try more challenging tasks. For the level 0 and level 1 interaction difficulties, all the participants were able to successfully complete them. However, level 2 two-way BCI appeared to be too difficult for them, where three out of five children presented a low degree of completion. This shows that a high learning cost is needed for BCI, especially compared with traditional interactions. It was difficult for the children to realize effective two-way BCI control in a short period. Therefore, in the formal experiments, the level 1 interaction mode was adopted.

### 4.2. Formal Experiments

In formal experiments, a series of contrast experiments was organized to test the three hypotheses proposed in Section 2. In particular, Conners Child Behavior Scores were adopted to assess children’s attention behaviors, and Schulte Grid Test was used to measure children’s concentration abilities. To test H1: There is no significant difference between subjects in attention behaviors before BCI training, non-parametric tests on independent samples were conducted. To test H2: The attention ability of subjects taking BCI training will improve significantly, and H3: The attention ability of subjects taking no BCI training will not significantly change, non-parametric tests on paired samples were deployed to examine the changes before and after training.

#### 4.2.1. Participants

**Figure 16:**
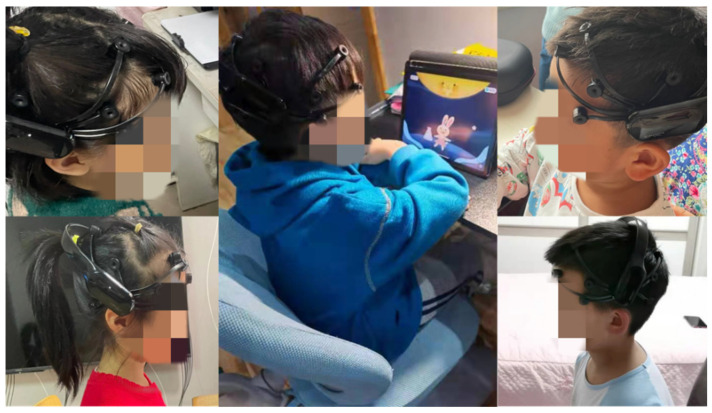
The game training process of the experimental group.

A total number of ten children were involved (as shown in Figure 16). They all met the characteristics of target users, namely, aged 4–6 years old, having previous experience with tablet games but no BCI experience. They were randomly divided into two groups: five in the experimental group (3 boys and 2 girls), and five in the control group (3 boys and 2 girls).

#### 4.2.2. Experiment Process

**Figure 17:**
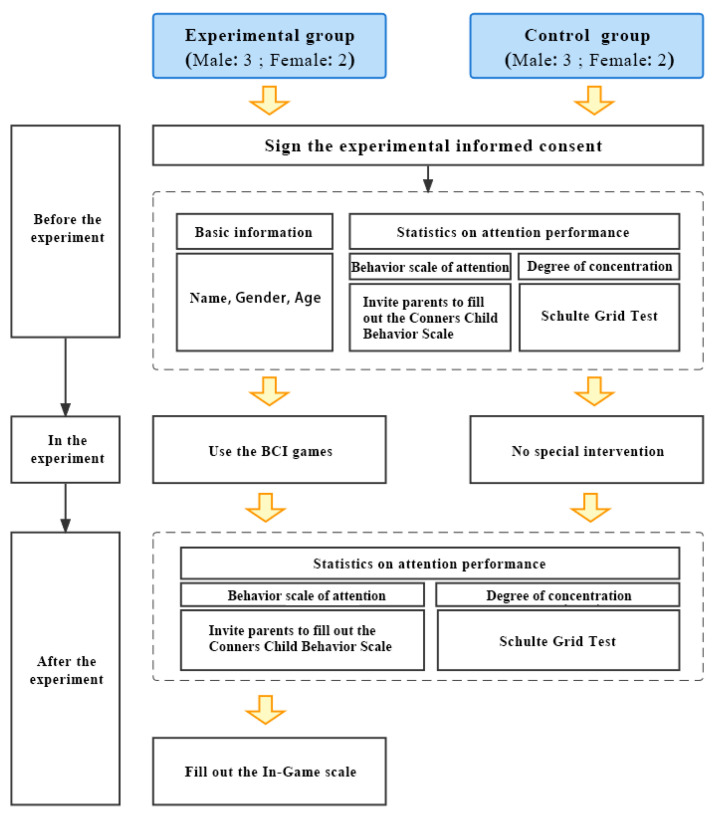
Experiment process.

The experimental process lasted 16 days and consisted of three parts (as shown in Figure 17).

Before the experiment, the basic information of all participants was collected. Their attention behaviors were investigated using special scales. For example, the Conners Child Behavior Scale (parent edition) was used to evaluate the behavioral symptoms in multiple aspects, such as learning behaviors, problem solving behaviors, impulsivity performance and so on. In terms of attention ability, the Schulte Grid Test was adopted to measure children’s concentration levels. With the preliminary investigation, a basic understanding of the participants in their daily performance and original attention ability was obtained.

In the experiment stage, the experiment group took the attention training every 2 days. They were guided to wear the BCI device, and system debugging was performed to ensure the good quality of signals. Subjects tried all three training games every time, and for each game, the training time was controlled at 5 min. A 3-min rest was arranged between two games. The attention performance during the whole training was observed and the EEG data were completely recorded. The control group received no special attention intervention during this period.

After the training experiment, the attention performance and concentration ability of the two groups were investigated again. By comparing children’s performance between experimental and control groups, and the attention ability before and after the training for the experimental group, the training effect could be statistically validated. In addition, the training experience was examined through Game Experience Questionnaire (GEQ) [60], and in-depth interviews were conducted for a better understanding of the participants’ training experience.

#### 4.2.3. Evaluation Metrics

In children’s behavior evaluation, the Conners Child Behavior Scale (Parent Edition) [61] was used. It analyzes common behavioral symptoms in children from the parent side, and is suitable for children and adolescents aged between 3 and 17. The most widely used is the revised version from 1978, and also adopted in this work. It involves 48 relevant questions divided into six factors, including peer relations, executive functioning, learning problems, hyperactivity/impulsivity, aggression and inattention. The four-point rating scale of 0, 1, 2, 3 (annotated by “Never”, “Sometimes”, “Often”, “Very often”) was used.

To assess children’s concentration level, the Schulte Grid Test was adopted. Basically, the Schulte Grid [62] is an experimental tool for conducting the Schulte Grid Test to measure the level of concentration. In the conventional Schulte Grid Test, 25 squares of 5 × 5 size are randomly distributed with numbers from 1 to 25, and the participant has to point out the positions of the numbers from 1 to 25 in order with his or her finger. The shorter the time they use to complete the test, the higher the concentration level they have. Considering the young age of the children, a 4 × 4 size Schulte Grid Test was adopted in this experiment.

For user experience evaluation, In-Game Experience scale was provided. It is a streamlined version of the GEQ [60], which is usually used to assess players’ game experience. The scale consists of seven dimensions: immersion, tension, competence, challenge, flow, and positive and negative affect, with two questions under each dimension, totaling 14 questions. Players need to fill in their agreement levels for the 14 statements according to their real game experience [63]. In this work, the parents helped the children in understanding and feeding back their training experience.

## 5. Results Analysis

Firstly, participants’ original attention ability in two groups before the training was analyzed. Considering that the sample size was very small, a non-parametric test was adopted. With their attention evaluation results from the Conners Child Behavior Scale and Schulte Grid Test, the Mann–Whitney test was carried out to make the comparison, as shown in Table 1. It can be seen that all the *p*-values of the six factors in the scale (namely peer relations, executive functioning, learning problems, hyperactivity/impulsivity, aggression and inattention) are larger than 0.05, which means there is no significant difference between the two groups in their original attention behaviors. H1 is well supported. In addition, the average scores of peer relations, executive functioning, learning problems and aggression are low, suggesting that no severe attention problem exists among these participants. The two groups have generally normal behavioral characteristics at this stage and can be further compared.

Regarding their concentration ability, the Mann–Whitney test was also applied on the Schulte Grid Test results between the experimental group and the control group. The results are listed in Table 2. It can be found that the p-value is 0.548, which is greater than 0.05, suggesting that there is no significant difference between the two groups. Therefore, no significant difference is presented in their original concentration ability, and the subsequent comparison of their training results has analytical significance.

### 5.1. Analysis of the Training Results before and after the Experiment

#### 5.1.1. Comparative Analysis before and after Training in Experimental Group

Wilcoxon test was conducted on the Conners Child Behavior Scale results in the experimental group before and after the experiment (as shown in Table 3). The results show that after the BCI training, the scores of the five factors in terms of peer relations, learning problems, aggression, inattention and the hyperactivity/impulsivity index decreased. The *p*-value of inattention is less than 0.05, which suggests a statistically significant change. The *p*-value of the hyperactivity/impulsivity index is 0.066, which may indicate a marginally significant change. Therefore, it can be understood that there is a certain positive influence on children’s attention behavior from the parent’s perspective, and the training game has a certain intervention effect on the children’s attention behaviors. H2 is partially supported.

Regarding the concentration ability, the Wilcoxon test was performed on the Schulte Grid Test results (as shown in Table 4). It was found that the children’s test time decreased after the training, and the P value was 0.043, less than 0.05. It suggests a significant improvement in the attention test after taking the training, and a basic conclusion can be reached that the BCI training can effectively improve the participants’ concentration ability.

#### 5.1.2. Comparative Analysis before and after Training in Control Group

In a similar way, the attention behaviors and concentration abilities of the control group were analyzed. The Wilcoxon test was also applied on the Conners Child Behavior Scale results and Schulte Grid Test results. The results are presented in Table 5 and Table 6. It can be found that all the *p*-values are greater than 0.05, which means there is no significant difference before and after the training. It further confirms that the attention level of the participants would not change significantly during the 16 days without any intervention. H3 is well supported.

According to the analysis above, it can be concluded that the experimental group with BCI training showed significantly improved attention levels. The training game could provide a positive intervention effect on children’s attention performance.

### 5.2. Analysis of the Game Reaction Time

To further analyze participants’ performance in each attention network, the participants’ reaction time (RT), which is the main parameter to measure the efficiency of each network, was emphatically analyzed. In particular, the change rate of the participants’ RT between the first-time training and last training was calculated. The rate of change = (RT (first time) − RT (last time))/RT (first time). The positive change rate means an improvement was presented. The higher the absolute value of the change rate, the better the improvement effect is.

**Figure 18:**
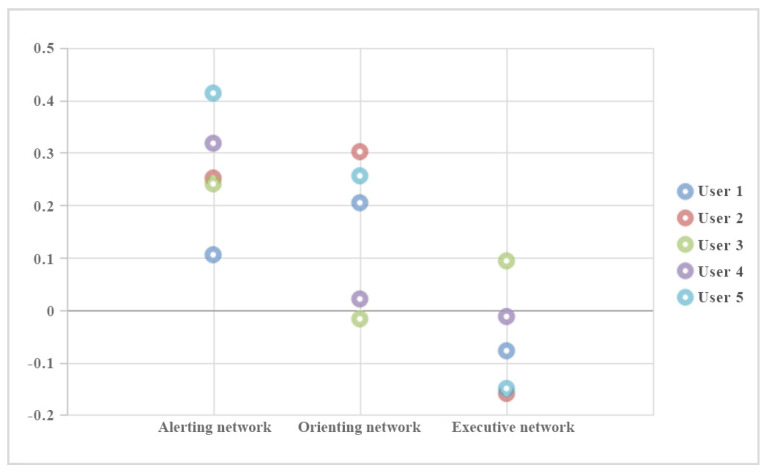
The rate of change of the attention network parameters.

As shown in Figure 18, positive change can be observed among the five users in the alerting and orienting networks. The change rates of the participants are mostly negative in the executive network. It can be explained from several possible aspects. First, the executive network involves many functions and is mainly for resolving reaction conflicts and self-regulation. It requires participants’ comprehensive cognitive reaction and is not easy to be trained by single-target training tasks. Second, the task design in the current training system is relatively simple to better fit young children. It is difficult to balance the complexity and adequacy in presenting incongruent and congruent network situations. Therefore, the task settings and game rhythm may need to be further improved.

### 5.3. Analysis of Game Experience

As shown in Table 7, the overall game experience is acceptable. The highest evaluation score is positive emotions (average score: 3.6 out of 4) and the negative emotion is low (average score: 1.3 out of 4). The two evaluation scores verify that the feedback from the participants are consistent, and the rating results are reliable. From the emotion-related scores, it can be seen that users enjoyed the training process very much, and clear positive feelings can be observed. The ratings on immersion and fluency are also good (higher than 3.0), which reflects that the game can create an immersive training environment and deliver a smooth game process for the participants. The ratings of competence and challenge are acceptable, which means the training has proper difficulty since the participants can have basic control over the tasks and feel challenged to some extent. The feedback on tension is low (with a score of 1.0), which implies the overall training is comfortable, and more inspiring challenges can be considered.

## 6. Discussion

In this work, a BCI was explored to support children’s attention training. Compared with existing attention training tools that are often targeted at children with ADHD for rehabilitation purposes, this work aims to discuss the possibility of home training with consumer-grade devices. Actually, attention training is needed not only for patients with ADHD but also for other wider populations, such as preschool children who need to prepare for systematic school learning.

For the specific settings of this training system, a compromise strategy was considered in the operation modes. For existing BCI video games, such as The Harvest Challenge [64], a BCI is not only used as an input device, but also the tool to monitor neurophysiological signals. Similarly, the OpenViBe2 movement platform includes many games and experiments based on the neuroscience BCI system [8]. For such BCI platforms, users must focus on the game or video and maintain their maximum attention. Actually, it is difficult to control the game using only EEG for most children [31]. It will lead to a heavy cognition burden and impact their training adherence. In addition, existing training systems lack considerations of user experience, so they are not very immersive and interesting [14,59,65]. Therefore, the integration of traditional interaction (e.g., screen touch, mouse click) and BCI control was proposed in this work. The participants can choose the proper interaction mode according to their attention situation, and make necessary adjustments after a period of training. With such settings, the training system was evaluated by the participants to have better immersion and user-friendliness.

In determining the proper thresholds for different task difficulties, it requires a reliable training database, and then to segment the low, medium and high attention levels. However, people’s attention abilities vary greatly, and the required sample size might be very large. Therefore, the precise estimation of the general attention situation of target participants is challenging. In this work, the same group of subjects were recruited to build their personal training data and then set the corresponding task difficulties according to their own attention performance. The attendant problem is that the training data cannot be used by other participants. Therefore, the corresponding attention difficulty thresholds need to be re-determined.

According to the main findings of this work, BCI training games can improve children’s attention performance. This finding is generally consistent with existing studies. For example, Reference [66] showed that EEG-based attention training could significantly improve children’s reading engagement and concentration levels, and achieve high training satisfaction and interest. Especially in the dimensions of hyperactivity, impulsivity and inattention, [65] also confirmed that BCI training can improve these aspects and overall attention levels. However, it is worth noting that existing studies mostly focus on ADHD children, while the proposed research is currently intended for attention improvement in normal children. Considering that children with ADHD have lower attention abilities, adaptive adjustment and task design improvement should be considered if the current training system is extended to wider applications, such as the training for children with ADHD or autism.

## 7. Conclusions

Generally, a systematic training system development approach was proposed based on the attention network and gamification design framework. Following the attention network structures, three types of training games were developed to offer the alerting, orienting and executive network tasks. Referring to the Octalysis gamification design framework, the multimodal game design elements, including visual interface, sound effects and integrated interaction modes, were accordingly developed. With the help of these theories, the training system is expected to provide a scientific training process and improve the training experience and adherence.

To validate the real training effect of the proposed system, a series of user experiments were conducted. Primarily, participants’ training was organized to build the EEG training data for attention level determinations. Then formal experiments were conducted to test the proposed training system. The results revealed that the training system can effectively create an immersive, inspiring and smooth training environment, and help the participants achieve better attention performance.

Specifically, the study makes several contributions as follows:

(i) An ANT-inspired training task construction process, which can guide task generation for altering, orienting and executive attentions.
(ii) A multimodal gamification design approach, which can guide the integration of multimodal design elements for improving user experience and training adherence.
(iii) A hypothesis-testing approach to empirically reveal the BCI training effect in improving children’s attention ability.

Generally, valuable references are provided for BCI attention training practices in designing effective and attractive training tasks, and for systematic evaluation of the training effect. Especially in the context of school education, the training system can provide an enjoyable way to help young students improve their attention level and better prepare for systematic school learning. Moreover, it can be a possible way for the school and teachers to measure and record students’ attention status, and to help with the formative analysis of students’ learning process. A better understanding of young children’s attention performance can be expected, which can further facilitate more targeted support of children’s attention and cognitive development. However, limitations still exist in this work. As a primary exploration, the sample size of the training experiments is very small so that the experiment reliability is limited. In the future study, more participants will be involved to further demonstrate the training effect. Moreover, the final adopted interaction mode is one-way BCI and traditional click, as pure brain control seems too hard for young children. To better investigate the BCI intervention influence, long-term training will be considered to help participants achieve basic learning of brain–computer interaction, and to then arrange the training tasks with complete BCI control to investigate if it can lead to a higher training efficiency and better training effect.

## Figures and Tables

**Table 1:** Conners Child Behavior Scale score comparison between the experimental group and the control group before the experiment.

Item	Experimental Group (N = 5) (M ± SD)	Control Group (N = 5) (M ± SD)	*p*
Peer relations	0.750 ± 0.186	0.664 ± 0.294	0.690
Executive functioning	0.050 ± 0.112	0.040 ± 0.089	1.000
Learning problems	0.550 ± 0.274	0.700 ± 0.671	0.841
Hyperactivity/Impulsivity	1.250 ± 0.354	1.050 ± 0.326	0.421
Aggression	0.700 ± 0.481	0.550 ± 0.209	0.841
Inattention	1.040 ± 0.305	0.900 ± 0.412	0.421

**Table 2:** The Schulte grid test comparison between the experimental group and the control group before the experiment.

Item	Experimental Group (N = 5) (M ± SD)	Control Group (N = 5) (M ± SD)	*p*
The time of the Schulte grid test	27.513 ± 2.342	26.495 ± 4.165	0.548

**Table 3:** The Conners Child Behavior Scores of the experimental group before and after the experiment.

Item	Before the Experiment (M ± SD)	After the Experiment (M ± SD)	*p*
Peer relations	0.750 ± 0.186	0.634 ± 0.096	0.197
Executive functioning	0.050 ± 0.112	0.080 ± 0.110	1.000
Learning problems	0.550 ± 0.274	0.500 ± 0.177	0.317
Hyperactivity/Impulsivity	1.250 ± 0.354	0.700 ± 0.209	0.066
Aggression	0.700 ± 0.481	0.500 ± 0.354	0.257
Inattention	1.040 ± 0.305	0.700 ± 0.100	0.043 *

* denotes significant influence with *p* < 0.05

**Table 4:** The Schulte Grid Test results before and after the experiment in the experimental group.

Item	Before the Experiment (M ± SD)	After the Experiment (M ± SD)	*p*
The time of the Schulte grid test	27.513 ± 2.342	24.995 ± 2.634	0.043 *

* denotes significant influence with *p* < 0.05

**Table 5:** The Conners Child Behavior Scores in the control group before and after the experiment.

Item	Before the Experiment (M ± SD)	After the Experiment (M ± SD)	*p*
Peer relations	0.664 ± 0.294	0.550 ± 0.373	0.273
Executive functioning	0.040 ± 0.089	0.240 ± 0.329	0.180
Learning problems	0.700 ± 0.671	0.850 ± 0.652	0.680
Hyperactivity/Impulsivity	1.050 ± 0.326	0.950 ± 0.647	0.854
Aggression	0.550 ± 0.209	0.500 ± 0.177	0.317
Inattention	0.900 ± 0.412	0.920 ± 0.559	0.892

**Table 6:** The Schulte Grid Test results before and after the experiment in the control group.

Item	Before the Experiment (M ± SD)	After the Experiment (M ± SD)	*p*
The time of the Schulte grid test	26.495 ± 4.165	25.838 ± 3.553	0.345

**Table 7:** The score of each dimension of the game experience scale.

Number	Item	M	SD
1	Immersion	3.30	0.447
2	Fluency	3.30	0.447
3	Competence	2.90	0.548
4	Positive emotions	3.60	0.224
5	Challenge	2.90	0.418
6	Negative emotions	1.30	0.447
7	Tension	1.00	0.612

## Data Availability

Not applicable.
